# Relative survival analysis of Merkel cell carcinoma across intersectional demographics using the Surveillance, Epidemiology, and End Results (SEER) database

**DOI:** 10.1016/j.jdin.2025.10.015

**Published:** 2025-11-03

**Authors:** Benjamin D. Hu, Brandon R. Block, Grace Rabinowitz, Raphaella Lambert, Andrew Zhu, Hannah Verma, Jón Gunnlaugur Jónasson, Bardur Sigurgeirsson, Shane Meehan, Jonathan Ungar, Jesse M. Lewin, Nicholas Gulati, Jonas A. Adalsteinsson

**Affiliations:** a Kimberly and Eric J. Waldman Department of Dermatology, Icahn School of Medicine at Mount Sinai, New York, New York; b Faculty of Medicine, University of Iceland, Reykjavik, Iceland; c Department of Pathology, Landspitali – The National University Hospital of Iceland, Reykjavik, Iceland

**Keywords:** Merkel cell carcinoma, population study, relative survival, SEER

*To the Editor:* Merkel cell carcinoma (MCC) is a rare, highly aggressive, cutaneous neuroendocrine malignancy.^1^ Its effect on survival across the intersections of sociodemographic factors such as age, race, and sex has not been well-explored with relative survival. Relative survival compares cancer patients’ survival with matched cancer-free individuals, minimizing non-cancer mortality differences. Unlike traditional models, RSRs avoid cause-of-death coding, more accurately estimating disease-attributable excess mortality across diverse subpopulations.^2^

Using the Surveillance, Epidemiology, and End Results (SEER) database, covering ∼48% of the U.S. population, we identified 7728 patients with MCC diagnosed from 2000 to 2021 and calculated age-standardized 5-year RSRs with 95% log-log confidence intervals (CIs), stratified by the intersections of age (15-44, 45-54, 55-64, 65-74, and 75+), race/ethnicity (non-Hispanic [NH] white, NH Black, NH Asian American and Pacific Islander, NH Native American, and Hispanic), and sex (Supplementary Table I, available via Mendeley at https://data.mendeley.com/datasets/vrtvpjbwgs/1).

Overall 5-year relative survival differed significantly from expected survival, with MCC patients having an RSR of 67.0% (CI = [65.4%, 68.5%]). Men had significantly worse relative survival than women (62.0% [59.9%, 64.0%] vs 74.7% [72.3%, 76.9%]). RSRs remained generally stable across ages <75 (70.0% to 74.9%) but decreased significantly for patients 75+ (56.6%). Stratifying by both sex and age, RSRs declined significantly for patients 75+ in men and women, with RSRs of 50.9% (47.3%, 54.3%) and 64.0% (59.7%, 67.9%), respectively (Fig 1). At all ages, men experienced larger survival impacts.

**Fig 1:**
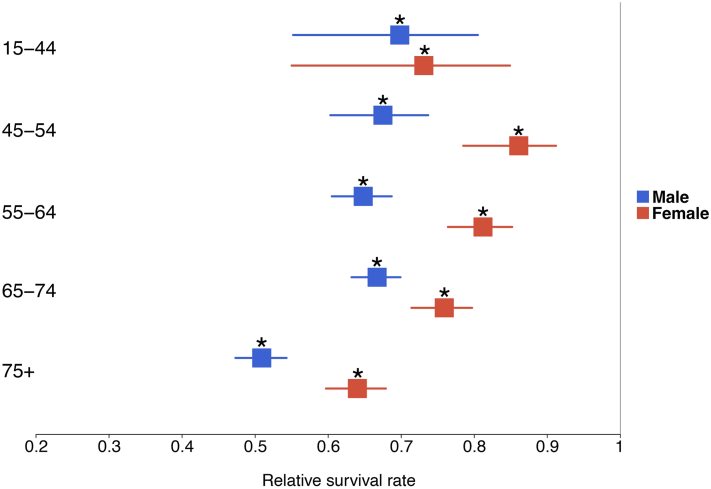
Relative 5-year survival in % and 95% log-log confidence intervals (CIs) for patients with Merkel cell carcinoma, split by age group and sex. ∗ indicates a significant difference in survival from subgroup counterparts without the disease. SEER∗Stat software does not calculate CIs in instances where relative survival range extends beyond 100%.

By race/ethnicity, Black patients had the lowest RSR (57.0% [42.6%, 66.1%]), while Asian, Hispanic, and White patients had RSRs ranging from 66.4% to 69.9%. Race-sex intersections revealed distinct patterns (Fig 2): RSRs among men were similar across race (61.1%-66.2%). In contrast, White, Asian, and Hispanic women had similar RSRs (72.0%-75.3%), but Black women had a significantly lower RSR of 52.5% (32.9%, 68.8%), the lowest of every race-sex combination.

**Fig 2:**
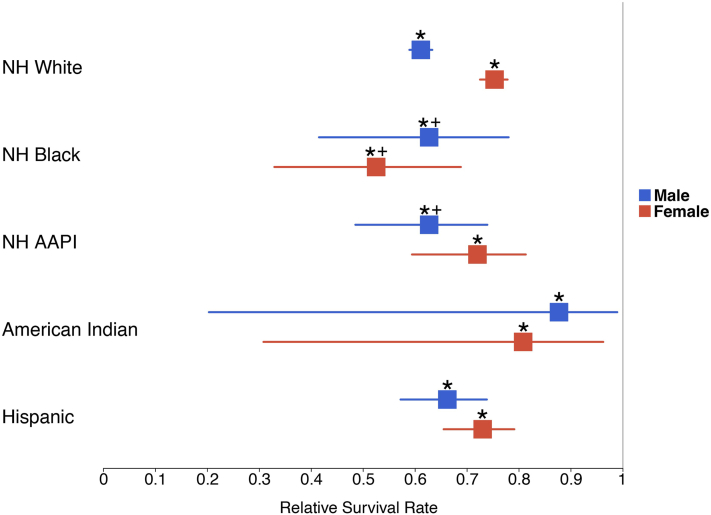
Relative 5-year survival in % and 95% log-log confidence intervals (CIs) for patients with Merkel cell carcinoma, split by race/ethnicity and sex. ∗ indicates a significant difference in survival from subgroup counterparts without the disease. SEER∗Stat software does not calculate CIs in instances where relative survival range extends beyond 100%. + indicates lack of age standardization by SEER∗Stat software due to insufficient data.

Our relative survival analysis underscores how intersectional demographics shape survival outcomes in MCC patients. Although incidence rises with age, the sharp decline in RSR in both men and women 75+ highlights a disproportionate mortality impact on older adults, a point not previously reported. One hypothesis is etiologic heterogeneity: older individuals may develop either ultraviolet-driven or polyomavirus-associated MCC, whereas younger patients—having less cumulative sun exposure—may more often develop polyomavirus-associated MCC.^3^^,^^4^

Furthermore, while it is well-known that men generally have worse survival than women, our analysis demonstrated Black women having the poorest RSR out of every race-sex combination, including all men, a finding not previously reported. Prior debate/conflicts over higher MCC mortality in Black patients, sometimes attributed to potential polyomavirus infection predominance, may stem from failure to account for sex-based survival differences.^5^

Limitations include smaller sample sizes from minority populations and certain race-sex subgroups not being age-standardized due to insufficient data (Fig 2). Sensitivity analyses using non-age-standardized data yielded the same directions and rankings of subgroup differences; notably, Black women remained the worst-surviving group.

In summary, MCC demonstrates significant survival impact, with certain intersectional race-sex and age-sex subgroups experiencing worse outcomes. These disparities highlight the need for equitable healthcare access, tailored awareness, and targeted research for specific subpopulations.

## Conflicts of interest

None disclosed.
